# Urinary ^1^H NMR Metabolomic Analysis of Prenatal Maternal Stress Due to a Natural Disaster Reveals Metabolic Risk Factors for Non-Communicable Diseases: The QF2011 Queensland Flood Study

**DOI:** 10.3390/metabo13040579

**Published:** 2023-04-21

**Authors:** Joshua P. Heynen, Rebecca R. McHugh, Naveenjyote S. Boora, Gabrielle Simcock, Sue Kildea, Marie-Paule Austin, David P. Laplante, Suzanne King, Tony Montina, Gerlinde A. S. Metz

**Affiliations:** 1 Canadian Centre for Behavioural Neuroscience, Department of Neuroscience, University of Lethbridge, 4401 University Drive, Lethbridge, AB T1K 3M4, Canada; josh.heynen@uleth.ca (J.P.H.); jsboora@student.ubc.ca (N.S.B.); 2 Southern Alberta Genome Sciences Centre, University of Lethbridge, 4401 University Drive, Lethbridge, AB T1K 3M4, Canada; 3 Department of Chemistry and Biochemistry, University of Lethbridge, 4401 University Drive, Lethbridge, AB T1K 3M4, Canada; rebecca.mchugh@uleth.ca; 4 Midwifery Research Unit, Mater Research Institute, University of Queensland, Brisbane, QLD 4072, Australia; g.simcock@psy.uq.edu.au (G.S.); sue.kildea@cdu.edu.au (S.K.); 5 School of Psychology, University of Queensland, Brisbane, QLD 4072, Australia; 6 Molly Wardaguga Research Centre, Faculty of Health, Charles Darwin University, Alice Springs, NT 0870, Australia; 7 Perinatal and Woman’s Health Unit, University of New South Wales, Sydney, NSW 2052, Australia; m.austin@unsw.edu.au; 8 Centre for Child Development and Mental Health, Lady Davis Institute for Medical Research, Jewish General Hospital, 4335 Chemin de la Côte-Sainte-Catherine, Montreal, QC H3T 1E4, Canada; david.laplante@ladydavis.ca; 9 Department of Psychiatry, Douglas Mental Health University Institute, McGill University, 6875 LaSalle Boulevard, Montreal, QC H4H 1R3, Canada; suzanne.king@mcgill.ca

**Keywords:** urine, objective hardship, subjective distress, child development, ancestral stress, risk prediction, developmental origins of health and disease (DOHaD), diabetes

## Abstract

Prenatal stress alters fetal programming, potentially predisposing the ensuing offspring to long-term adverse health outcomes. To gain insight into environmental influences on fetal development, this QF2011 study evaluated the urinary metabolomes of 4-year-old children (*n* = 89) who were exposed to the 2011 Queensland flood in utero. Proton nuclear magnetic resonance spectroscopy was used to analyze urinary metabolic fingerprints based on maternal levels of objective hardship and subjective distress resulting from the natural disaster. In both males and females, differences were observed between high and low levels of maternal objective hardship and maternal subjective distress groups. Greater prenatal stress exposure was associated with alterations in metabolites associated with protein synthesis, energy metabolism, and carbohydrate metabolism. These alterations suggest profound changes in oxidative and antioxidative pathways that may indicate a higher risk for chronic non-communicable diseases such obesity, insulin resistance, and diabetes, as well as mental illnesses, including depression and schizophrenia. Thus, prenatal stress-associated metabolic biomarkers may provide early predictors of lifetime health trajectories, and potentially serve as prognostic markers for therapeutic strategies in mitigating adverse health outcomes.

## 1. Introduction

Human populations are increasingly exposed to physical and psychological stress associated with climate change, including natural disasters, forced migration, starvation, or exposure to environmental toxins [1,2,3]. If pregnant mothers are exposed to these adverse events, the ensuing stress response may induce excessive glucocorticoid release that has the potential to affect the health of the developing fetus [4,5,6]. Accordingly, prenatal maternal stress (PNMS) has been shown to affect birth outcomes and potentially predispose the child to adverse mental and physical health later in life [1,3,7]. For example, children who experienced greater maternal stress from the 2008 Iowa Flood in utero displayed higher body mass index (BMI) and increased risks for adiposity at age 2.5 years [8,9], along with higher cortisol reactivity [10]. Similar results were also obtained due to prenatal exposure to high maternal stress caused by the 1998 Quebec Ice Storm, with the children displaying higher BMI and adiposity at ages 5.5, 8.5, 13.5, and 15.5 years [11], poorer language abilities [12], and altered testosterone and cortisol reactivity [13]. Notably, the mother’s objective hardship and her subjective experience are associated with different outcomes. Greater maternal subjective distress was associated with diminished cognitive functioning in the toddler, whereas both objective hardship and subjective distress were linked to reduced language-related development in children who experienced the 2008 Iowa Flood in utero [14]. Furthermore, exposure to high objective hardship during the 1998 Quebec Ice Storm was associated with lower intelligence quotients at 2 and 5.5 years of age [12,15].

The transmission of maternal stress to the fetus is complex and multifaceted. The placenta represents a protective barrier to the transmission of maternal stress to the fetus. During gestation, the placenta and fetal brain display an overexpression of the enzyme 11β-hydroxysteroid dehydrogenase (11β-HSD) type 2 [16,17,18]. 11β-HSD type 2 converts cortisol to cortisone [19], thereby protecting the developing fetus from the growth-inhibiting and pro-apoptotic effects of cortisol. Placental impairment in adaptive regulation of 11β-HSD expression in response to stress [20] has been associated with growth restriction of the infant and the development of psychopathologies in later life, including anxiety traits and schizophrenia [18,21,22,23,24]. Thus, maternal stress may alter fetal development via changes in the placenta, a process known as fetal programming [25].

Importantly, outcomes linked to severe PNMS can last into adolescence and adulthood. For example, individuals who were prenatally exposed to the 1976 Tangshan earthquake had a significantly higher incidence of depression in adulthood [26]. Prenatal exposure to earthquakes was also shown to raise the risk of schizophrenia spectrum disorders [27]. Moreover, the increased severity of maternal stress during pregnancy caused by the 1998 Quebec Ice Storm was associated with greater offspring insulin secretion in adolescence, suggesting a potential risk of insulin resistance and metabolic disease [28]. This finding was also supported by urinary metabolomic analysis of offspring exposed to the 1998 Quebec Ice Storm in utero, suggesting a potential risk for type 2 diabetes mellitus [29]. Indeed, one retrospective study found that maternal bereavement during pregnancy was significantly associated with offspring development of type 2 diabetes mellitus in adulthood [30]. Notably, the risk for metabolic disease shows sex-specific programming [31].

A series of floods impacted Queensland, Australia, beginning in November 2010 and lasting until January 2011. The 2011 Queensland Flood (QF2011) Australian study commenced following significant flooding that forced thousands to evacuate their homes, caused more than $2.3 billion AUD in reported damages, and resulted in 33 deaths [4]. The 2011 Queensland Flood represented an environmental stressor with a measurable objective and subjective impacts on pregnant women [4]. Previous work on the QF2011 cohort found that high maternal subjective distress in later pregnancy significantly reduced placental corticotropin-releasing hormone (CRH) concentrations in girls only, indicating sex-specific influences [31]. Importantly, higher CRH has been linked to reduced fetal adaptation to the uterine environment, resulting in lower birth weight and slower fetal growth [1]. In addition, work on the same study found an association between low levels of maternal hardship, high hair copper levels, and language developmental deficits in 4-year-old boys [32].

Differences in the outcomes of PNMS may vary due to changes in placental function [33] and an altered metabolome [5]. Metabolic profiling via proton nuclear magnetic resonance (^1^H NMR) spectroscopy has emerged as a particularly useful tool for characterizing pathophysiological consequences of fetal-environment interactions [29,34,35,36]. Urinary metabolic profiles represent the downstream physiological consequences of these interactions [37]. Although ^1^H NMR spectroscopy is less sensitive (micromolar concentrations) than other metabolomics approaches, such as liquid or gas chromatography-mass spectroscopy (nanomolar concentrations), NMR spectroscopy is advantageous when working with samples of limited availability. As ^1^H NMR is non-destructive, samples can be reused for additional analyses using other methods. Furthermore, ^1^H NMR spectroscopy is a high-throughput technique that requires minimal sample preparation and provides access to quantify the most metabolites in urine when compared to mass-spectroscopy based techniques (209 vs. 179, respectively) [37].

The present study utilized ^1^H NMR to investigate the metabolic alterations arising from PNMS in urine. The analysis of males and females was carried out separately, as there are significant sex differences in the urine metabolome [38]. Although PNMS is known to impact offspring development and life-long health risks [7,39], the mechanisms by which these changes occur remain unclear [40,41,42]. By including objective hardship as a stressor, this study considers stress independently of parental influences or family characteristics such as socio-economic status. This feature facilitates the ability to determine how objective and subjective factors, separately or synergistically, influence child development. Our aim was to identify metabolic profiles in male and female offspring that reflect their mother’s levels of objective hardship and subjective distress in relation to the 2011 Queensland Flood and provide insight into potential long-term health risks and their mechanisms of action. Overall, this study highlights the potential role of maternal experience in shaping the metabolic responses in children and suggests potential targets for early diagnosis, intervention, and prevention strategies.

## 2. Materials and Methods

### 2.1. Study Design

#### 2.1.1. Post-Flood Recruitment

In January 2011 Queensland, Australia experienced its worst flooding in 30 years. The recruitment of study participants began once ethics approval was obtained in April 2011 and continued up to 12 months post-flood with full details outlined in the protocol [4]. Many participants were re-recruited from an unrelated study (the M@NGO study; [43,44]) at the Mater Mothers Hospital, while others were recruited by their midwives, or by research assistants, or responded to flyers at the hospital. Women included in the study were 18 years of age or older, residing in the area of Brisbane on 10 January 2011, spoke fluent English, and were pregnant or became pregnant within days of peak flooding with a single offspring. All 230 women had given birth within the first 12 months after the beginning of the flood [45]. Mothers’ mean age at childbirth was 31.12 years (SD = 5.28; range = 19.52–47.33) and their mean socio-economic status was 1050.77 (SD = 59.13) [46]. Maternal socio-economic status was determined using the Socio-Economic Index For Area (SEIFA) scores, and was based on Australian postcodes and census data. A detailed description of the study protocol can be found in King et al. [4], and maternal SES can be found in Chen et al. [46].

#### 2.1.2. Assessment of Objective Hardship and Composite Subjective Distress

Upon recruitment, the 230 participants were administered the QFOSS200 (Queensland Flood Objective Stress Survey), the IES-R (Impact of Events Scale—Revised; [47]), the Peritraumatic Distress Inventory (PDI; [48]), and the Peritraumatic Dissociative Experiences Questionnaire (PDEQ; [49]). Participants completed all surveys again at 12 months post-flood.

The QFOSS200 questionnaire assessed pregnant women’s flood-related objective hardship in terms of threat, loss, scope, and change. Each dimension had a maximum of 50 points for a total maximum score of 200 points, with higher scores reflecting greater objective hardship [4,45]. The QFOSS200 survey was based on an existing objective hardship survey used to assess the flood experiences of pregnant mothers during the 2008 Iowa flood [50]. The QFOSS200 survey was administered upon recruitment, and again 12 months post-flood to update participants’ experiences with insurance companies and financial loss [4].

The IES-R, PDI, and PDEQ were used to identify potential flood-related adverse mental health symptoms. The 10-item PDEQ assessed the mother’s dissociative-like experiences, whereas the 13-item PDI assessed panic-like reactions [51]. The PDI and PDEQ questionnaires were used to assess how the participants recalled having felt at the time of the flood. The IES-R is a 22-item assessment, commonly used to identify symptoms of post-traumatic stress disorder [47]. This questionnaire addressed how participants in the QF2011 study currently felt—at recruitment and 12 months post-flood—to assess the ongoing psychological impact resulting from the flood [4].

A composite score for mothers’ subjective distress (COSMOSS) was created utilizing Principal Component Analysis (PCA) of the three subjective distress scores, with the algorithm deriving: 0.358 × IESR + 0.397 × PDI + 0.387 × PDEQ. COSMOSS scores represent a variable derived from the PCA that described 76.27% of the total subjective distress variance, standardized to a mean of 0, and a standard deviation of 1.0 [45].

For the purposes of analyses in the present study, maternal scores were divided at the median into high vs. low objective hardship groups and high vs. low composite subjective distress groups.

### 2.2. Sample Collection and Preparation

Of the 230 mothers who took part in the original study, urine samples were only provided from 89 of the 4-year-old offspring (male: *n* = 50; female: *n* = 39) and these samples were collected at the first passage of the day and stored at −80 °C. Frozen samples were transported to the University of Lethbridge for metabolomic analysis. The following procedure was carried out for all 89 samples. A total of 160 µL of buffer, 40 µL of 0.02709% *w*/*v* D_2_O with trimethylsilyl propanoic acid (TSP), and 400 µL of urine was added to each microcentrifuge tube. The buffer consisted of a 4:1 ratio of dibasic potassium phosphate (K_2_HPO_4_) to monobasic potassium phosphate (KH_2_PO_4_) with a concentration of 0.625 M and contained 3.75 mM of NaN_3_ as an antimicrobial agent and 3.75 mM of potassium fluoride (KF). The centrifuge tube was then vortexed and centrifuged at 12,000 rpm at 4 °C for 5 min. A 550 µL aliquot of the resulting supernatant was transferred to an NMR tube. All samples were checked for a pH of 7.4 ± 0.05 before ^1^H NMR analysis.

### 2.3. NMR Data Acquisition

Spectra were collected on a 700 MHz Bruker Avance III HD spectrometer (Bruker Ltd., Milton, ON, Canada), using a 1-D NOESY gradient water suppression pulse sequence. Samples were run for 128 scans to a total acquisition size of 128 k, zero filled to 256 k, automatically phased, baseline corrected, and line-broadened by 0.3 Hz. The spectra were then exported to MATLAB (MathWorks, Natick, MA, USA) to undergo recursive segment wise peak alignment [52] and subsequently binned using Dynamic Adaptive Binning, followed by manual correction [53]. Prior to modeling, the data were normalized, log-transformed, and pareto-scaled [54]. All spectra were referenced to the TSP peak (0.00δ).

### 2.4. Statistical Analyses

A Shapiro-Wilk test was used to assess the normality of the data and all bins were determined to be non-parametric. The Wilcoxon-Mann-Whitney U (MW) test was employed following the result of non-parametric data [55]. All univariate statistical tests underwent Bonferroni-Holm correction to correct for multiple comparisons and a *p*-value equal to or less than 0.05 was considered significant. Variable Importance Analysis based on random Variable Combination (VIAVC) was utilized to identify significantly altered bins between groups for use in metabolite identification [56]. The VIAVC algorithm employs binary matrix resampling, ensuring each variable has an equal chance of being selected. The importance of each variable is assessed based on the percent increase or decrease in the area under the curve (AUC) corresponding to the receiver operator characteristic curve (ROC) when added or removed from the respective subsets. This process is repeated until only bins that result in the best class separation remain. Orthogonal Projection to Latent Structures—Discriminant Analysis (OPLS-DA), a supervised multivariate test [57], was carried out to maximise observed differences between high vs. low maternal composite subjective distress as well as high vs. low maternal objective hardship. All multivariate models were validated by double ten-fold cross-validation and permutation testing using 2000 permutations [58,59]. OPLS-DA analysis was carried out utilizing bins identified as significant by VIAVC, as they led to the most observed separation between comparison groups. Males and females were analyzed separately, as there are significant sex differences in the urinary metabolome [38].

### 2.5. Metabolite Identification and Pathway Analysis

Chenomx 8.2 NMR suite software (Chenomx Inc., Edmonton, AB, Canada), in conjunction with the Human Metabolome Database (HMDB) of urinary metabolites [60,61,62], was used to identify the metabolites corresponding to bins found to be significantly altered by either VIAVC or MW testing. Metaboanalyst was used to perform pathway topology analysis using the metabolites identified as significant by VIAVC. The homo sapiens pathway library was utilized for all comparisons, the hypergeometric test was selected for the over-representation analysis, and relative-betweenness centrality was chosen for the pathway topology analysis. Pathways with a *p*-value equal to or less than 0.05 were labeled and considered to be significant.

## 3. Results

### 3.1. Maternal Objective Hardship and Subjective Distress Produce Unique Metabolome Profiles

According to the median split of the QFOSS200 objective hardship questionnaire results, 26 of the 50 male offspring mothers experienced high objective hardship and the remaining 24 experienced low objective hardship. COSMOSS results showed that 25 mothers of male offspring had high composite subjective distress scores and 25 had low composite subjective distress scores.

QFOSS200 scores classified 20 of the 39 mothers of female offspring as having experienced high objective hardship, the remaining 19 were categorized as having experienced low objective hardship. COSMOSS data resulted in 23 mothers of females with high composite subjective distress, whereas the remaining 16 scored low composite subjective distress.

Comparisons were conducted separately for high versus low objective hardship in males, high versus low subjective distress in males, high versus low objective hardship in females, and high versus low subjective distress in females. Differences in urinary metabolite concentrations that were statistically significant between comparison groups were identified using VIAVC. Out of 382 bins, 26 unique metabolites previously found in urine were significantly altered in males associated with maternal objective hardship, whereas 13 were altered in males associated with maternal composite subjective distress (Table 1). Thirty-one metabolites were significantly altered in females because of maternal objective hardship, and twenty-five metabolites were altered in females when considering maternal composite subjective distress (Table 2) [61].

**Figure 1:**
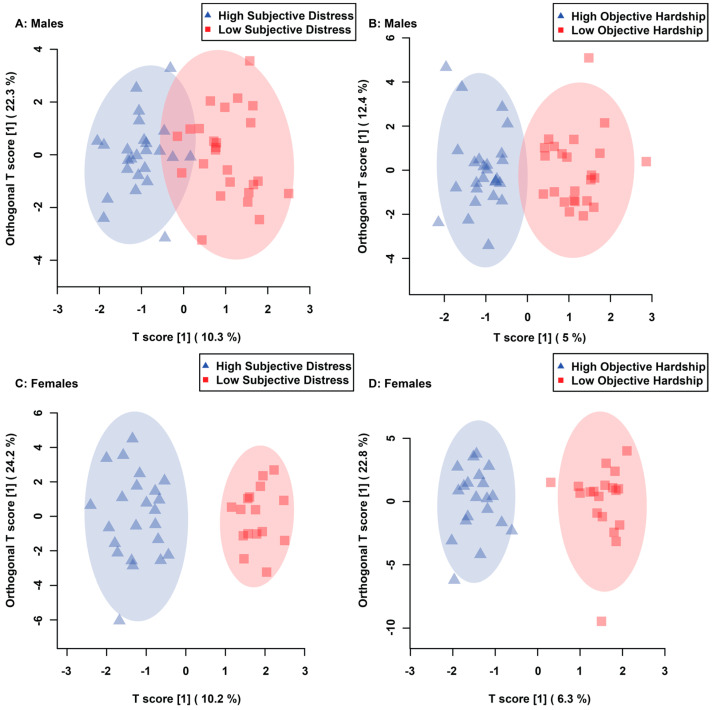
Orthogonal Projections to Latent Structures Discriminant Analysis (OPLS-DA) score plots representing the statistically significant supervised separation of maternal composite subjective distress in males (**A**), maternal objective hardship in males (**B**), maternal composite subjective distress in females (**C**) and maternal objective hardship in females (**D**) comparisons. Each triangle and square represent one individual, plotted using urinary metabolites found to be statistically significant by VIAVC testing. Shaded ellipses represent the 95% confidence interval. The x-axis represents between-group (predictive) variation, whereas the y-axis represents within-group (orthogonal) variation.

**Figure 2:**
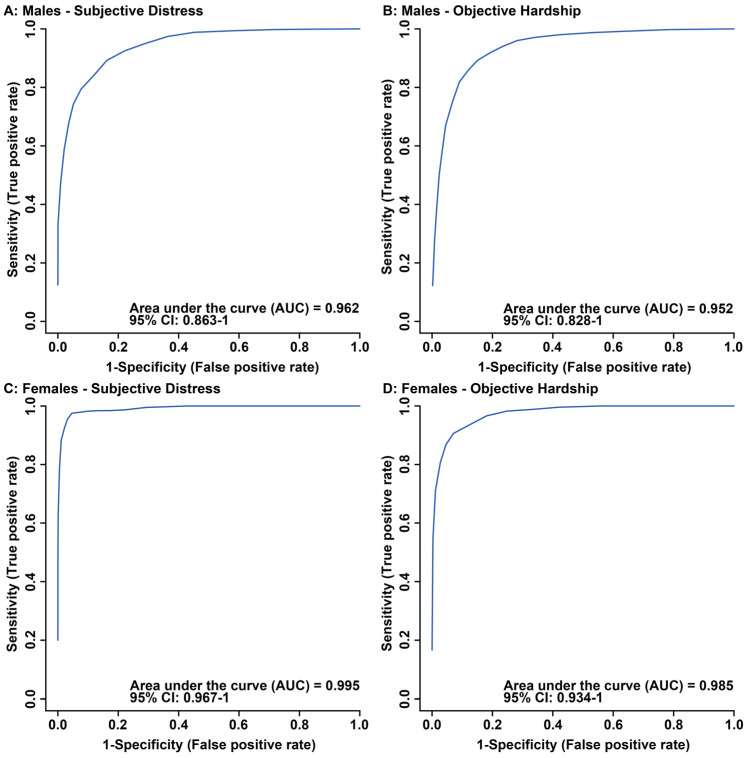
Receiver Operator Characteristic (ROC) curves of maternal composite subjective distress in males (**A**), maternal objective hardship in males (**B**), maternal composite subjective distress in females (**C**), and maternal objective hardship in females (**D**) comparisons. The area under the curve (AUC) and confidence interval (CI) corresponding to the comparison are indicated in each figure. Each figure was created utilizing metabolites identified as significant by VIAVC best subset.

OPLS-DA scores plots of males showed significant separation in maternal composite subjective distress and maternal objective hardship groups, when comparing median-split high vs. low scores (Figure 1A,B). Cross validation and permutation testing confirmed the observed supervised separation in maternal composite subjective distress (*p* = 0.00004, Q2: 0.594, R2: 0.757, Figure 1A) and objective hardship (*p* = 0.00004, Q2: 0.635, R2: 0.837, Figure 1B). ROC analysis validated the identified metabolites as contributing to alterations in males due to maternal composite subjective distress (AUC: 0.962, CI: 0.863–1, Predictive accuracy: 87.2%, Figure 2A) and objective hardship (AUC: 0.952, CI: 0.828–1, Predictive accuracy: 87.6%, Figure 2B) group comparisons. In females, supervised OPLS-DA scores plots display significant separation between high vs. low groupings for both composite subjective distress and objective hardship (Figure 1C and Figure 1D, respectively). Cross validation and permutation testing confirmed the observed supervised separation in both maternal composite subjective distress (*p* = 0.00004, Q2: 0.84, R2: 0.912, Figure 1C) and objective hardship (*p* = 0.00004, Q2: 0.791, R2: 0.935, Figure 1D). ROC analysis validated the identified metabolites as contributing to alterations in females due to maternal composite subjective distress (AUC: 0.995, CI: 0.967–1, Predictive accuracy: 97.2%, Figure 2C) and objective hardship group comparisons (AUC: 0.985, CI: 0.934–1, Predictive accuracy: 90.3%, Figure 2D).

### 3.2. Maternal Objective Hardship and Subjective Distress Program Protein Synthesis, Energy Metabolism, and Carbohydrate Metabolism

High maternal objective hardship in males downregulated 20/32 (62.5%) bins identified as significant by VIAVC (Table 1). In contrast, 9/14 (64%) significant bins were upregulated due to high maternal composite subjective distress in males (Table 1).

In females, 23/39 (58.9%) significant bins were upregulated due to high maternal objective hardship (Table 2), and 13/25 (52%) bins were downregulated in females of high maternal composite subjective distress (Table 2).

**Figure 3:**
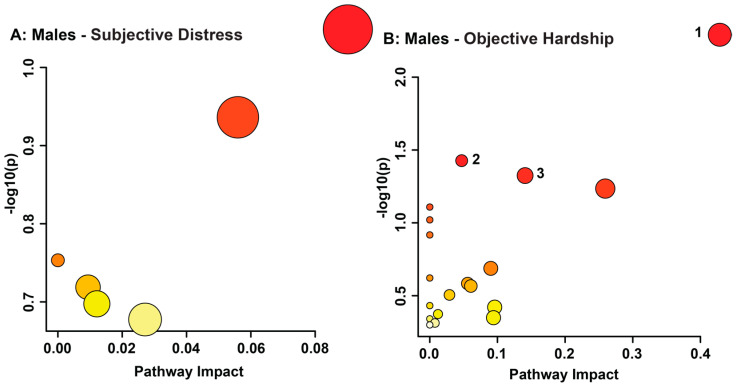
Metabolomic Pathway Topology Analysis of males whose mothers experienced composite subjective distress (**A**) and objective hardship (**B**) conducted utilizing bins identified as significantly altered by VIAVC. A higher value on the y-axis relates to a lower *p*-value, with 1.3 referring to a *p*-value of 0.05. The x-axis provides a measure of the predicted impact on the metabolic pathway due to the identified metabolites. The color of each circle is indicative of its *p*-value, whereas the size of each circle relates to its impact factor, with a darker color and larger circle indicating a lower *p*-value and greater impact factor, respectively. Only the pathways with a significant *p*-value (less than or equal to 0.05) are numbered and refer to the following metabolic pathways: (1) Taurine and hypotaurine metabolism; (2) Pentose phosphate pathway; and (3) Lysine degradation.

**Figure 4:**
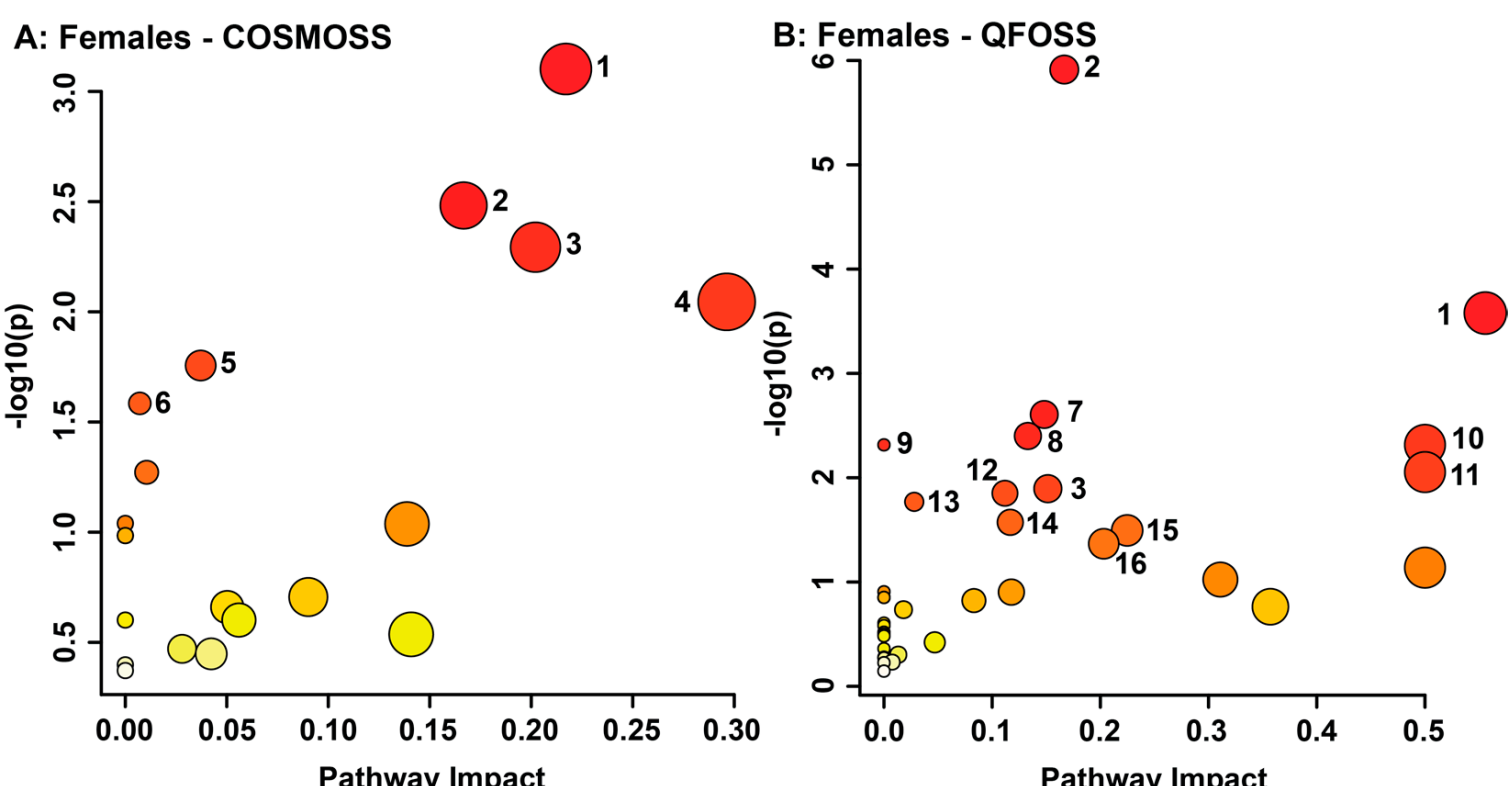
Metabolomic Pathway Topology Analysis of females whose mothers experienced composite subjective distress (**A**) and objective hardship (**B**) conducted utilizing bins identified as significantly altered by VIAVC. A higher value on the y-axis relates to a lower *p*-value, with 1.3 referring to a *p*-value of 0.05. The x-axis provides a measure of the predicted impact on the metabolic pathway due to the identified metabolites. The color of each circle is indicative of its *p*-value, whereas the size of each circle relates to its impact factor, with a darker color and larger circle indicating a lower *p*-value and greater impact factor, respectively. Only the pathways with a significant *p*-value (less than or equal to 0.05) are numbered and refer to the following metabolic pathways: (1) Glycine, serine, and threonine metabolism; (2) Aminoacyl-tRNA biosynthesis; (3) Galactose metabolism; (4) Cysteine and methionine metabolism; (5) Tyrosine metabolism; (6) Pantothenate and CoA biosynthesis; (7) Glyoxylate and dicarboxylate metabolism; (8) Starch and sucrose metabolism; (9) Nitrogen metabolism; (10) D-Glutamine and D-glutamate metabolism; (11) Ascorbate and aldarate metabolism; (12) Glutathione metabolism; (13) Porphyrin and chlorophyll metabolism; (14) Arginine biosynthesis; (15) Arginine and proline metabolism; (16) Pentose and glucuronate interconversions.

Changes in metabolite concentration deemed significant by exploratory statistical analysis were grouped by biological metabolic pathway. In order to predict the metabolic pathways that were potentially altered in both sexes due to the effects of maternal objective hardship and composite subjective distress, all significant metabolites identified by VIAVC were utilized (Figure 3 and Figure 4).

Maternal composite subjective distress in males did not indicate any metabolic pathway as potentially altered (Figure 3A, Table S1). Males’ maternal objective hardship pathway topology analysis revealed the taurine and hypotaurine metabolism (*p* = 0.0051158, impact = 0.42857), pentose phosphate pathway (*p* = 0.037427, impact = 0.04712), and lysine degradation (*p* = 0.047378, impact = 0.14085) as potentially altered metabolic pathways (Figure 3B, Table S1).

Maternal composite subjective distress in females presented potentially altered metabolic pathways including glycine, serine, and threonine metabolism (*p* = 0.00079075, impact = 0.21707); aminoacyl-tRNA biosynthesis (*p* = 0.003291, impact = 0.16667); galactose metabolism (*p* = 0.0050899, impact = 0.20208); cysteine and methionine metabolism (*p* = 0.009009, impact = 0.2963); tyrosine metabolism (*p* = 0.017523, impact = 0.03714); and pantothenate and CoA biosynthesis (*p* = 0.02603, impact = 0.00714; Figure 4A, Table S1). Pathway topology analysis for objective hardship in females showed the following metabolic pathways as potentially impacted: aminoacyl-tRNA biosynthesis (*p* = 1.22 × 10^−6^, impact = 0.16667); glycine, serine, and threonine metabolism (*p* = 0.00026396, impact = 0.55577); glyoxylate and dicarboxylate metabolism (*p* = 0.0024763, impact = 0.14815); starch and sucrose metabolism (*p* = 0.0039813, impact = 0.13303); nitrogen metabolism (*p* = 0.0048415, impact = 0); D-glutamine and D-glutamate metabolism (*p* = 0.0048415, impact = 0.5); ascorbate and aldarate metabolism (*p* = 0.0088294, impact = 0.5); galactose metabolism (*p* = 0.012742, impact = 0.15148); glutathione metabolism (*p* = 0.014093, impact = 0.11182); porphyrin and chlorophyll metabolism (*p* = 0.017032, impact = 0.02799); arginine biosynthesis (*p* = 0.026767, impact = 0.11675); arginine and proline metabolism (*p* = 0.032014, impact = 0.22479); and pentose and glucuronate interconversions (*p* = 0.042975, impact = 0.20312; Figure 4B, Table S1).

## 4. Discussion

Here we show that high and low flood-related composite subjective distress and objective hardship are associated with significant differences in offspring urinary metabolome measured at four years of age. This study revealed significant alterations in metabolic pathways linked to oxidative stress. Oxidative stress reflects an imbalance between reactive oxygen species (ROS) and antioxidants, leading to cellular apoptosis and damage to DNA and proteins. Furthermore, psychological distress in adults [63], as well as PNMS [64], can upregulate oxidative stress via the HPA axis, primarily through inflammatory cytokines [65]. Modulation of oxidative stress by psychological or physical stress has been linked to a greater imbalance in oxidation, thereby contributing to a higher risk for oxidative stress related diseases later in life [63,64,66]. Interestingly, many of the pathways involved in the antioxidant response play crucial roles in protein synthesis, energy metabolism, and carbohydrate metabolism. This study found significant alterations in antioxidant metabolic pathways including glutathione metabolism, taurine and hypotaurine metabolism, and ascorbate and aldarate metabolism, suggesting a link between early adverse experiences and the risk of metabolic illness later in life [67,68,69,70].

Cysteine, a non-essential amino acid important for protein synthesis, detoxification, and the energy metabolism, was significantly downregulated in males as a result of maternal objective hardship. Moreover, cysteine concentrations were reduced in females prenatally exposed to both high objective hardship and subjective distress. The cysteine and methionine metabolism, dysregulated in females with high maternal composite subjective distress, describes the synthesis of cysteine and its transfer to other metabolic pathways. Cysteine plays a vital role in the synthesis of key antioxidants, including glutathione and taurine, both a part of phase II metabolism [71,72]. The glutathione metabolism, part of the energy metabolism, was significantly altered in females with high maternal objective hardship and presented with a high impact in males prenatally exposed to high maternal objective hardship (*p* = 0.05819, impact = 0.25939). Glutathione was upregulated in males with high levels of maternal objective hardship and plays a vital role in the body’s toxic waste disposal by protecting against peroxidation and conjugation reactions by binding with toxic chemicals to detoxify and remove them from the body. Glutathione reduces to form cysteine-S-conjugates, such as cysteine-S-sulfate seen in both males and females with high maternal objective hardship, with ROS before being excreted through urine or bile [73]. Thus, cysteine concentrations may be lowered due to the increased synthesis of glutathione for detoxification, producing cysteine-S-conjugates. These findings suggest that glutathione metabolism is modulated by PNMS-related ROS in both sexes due to maternal objective hardship.

Taurine and hypotaurine metabolism presented as significant in males with high maternal objective hardship when compared to low maternal objective hardship. The upregulation of taurine and the downregulation of one of its components, cysteine, may result from oxidative stress due to toxicant insults in the liver [72,74]. Indeed, increases in urinary excretion of taurine are seen in many disease states related to oxidative stress, including diabetes, as well as cardiovascular diseases such as atherosclerosis [75,76,77]. In addition to taurine’s antioxidant properties, it may also play a role in modulating glutathione synthesis, preventing decreases in glutathione during oxidative stress [78]. The upregulation of taurine found in males of high maternal objective hardship may explain the increases in glutathione as well.

The ascorbate and aldarate metabolism and the pentose and glucuronate interconversions pathway, both significant in females with high maternal objective hardship, provide another form of phase II metabolism, glucuronidation. Glucuronidation occurs via the conversion of UDP-glucose into D-glucuronate, and functions to make a variety of substances, including toxicants and hormones, more water-soluble for transport or elimination from the body [79,80,81]. The significance of glucuronidation in females exposed to high maternal objective hardship implies increased detoxification occurring due to elevated ROS concentrations. The alterations observed in the taurine and hypotaurine metabolism and glutathione in males and ascorbate and aldarate metabolism in females suggest that gender may have a role in PNMS-related impacts on oxidative pathways. Indeed, significant gender differences in oxidative stress have been observed. Previous literature shows that males are considerably more susceptible to oxidative stress than females [82]. Our study mirrors this finding with males displaying upregulation of the antioxidant glutathione, but not females. Higher estrogen concentrations in females are thought to mediate their greater resilience to oxidative stress [82]. This may account for the significance of glucuronidation and glutathione metabolism in females only, providing a mechanism through which PNMS reduces the risk for oxidative stress-related disorders.

3-Chlorotyrosine, a potential marker for oxidative damage and inflammation, was significantly altered in males exposed to high maternal composite subjective distress and in both female comparisons. The only known producer of 3-chlorotyrosine in the human body is hypochlorous acid, which is produced by myeloperoxidases released by neutrophil degranulation [83,84]. Due to its link with immunity, 3-chlorotyrosine is present in those with atherogenesis, respiratory distress, as well as severely asthmatic patients [85,86]. Higher concentrations of 3-chlorotyrosine in infancy may serve as a potential biomarker for the future development of chronic lung diseases. Interestingly, exposure to prenatal and early life stress increases the risk for childhood respiratory disorders, suggesting a potential link [87,88,89]. The elevation of 3-chlorotyrosine as seen in this study is consistent with previous results of PNMS due to the 1998 Quebec Ice Storm [29].

The glycine, serine, and threonine metabolic pathway describes the process of serine’s conversion into cystathionine, cysteine, and glycine and was potentially altered in females prenatally exposed to high maternal objective hardship and subjective distress. This pathway, part of the energy metabolism, is frequently observed in stress-related studies [29,34,90,91,92]. Previous literature report that reduced glycine concentrations in plasma may often reflect elevated urinary glycine excretion [93]. Additionally, long-term insufficiencies in plasma glycine are a significant factor in the etiology of metabolic diseases [93]. These results reflect metabolic changes associated with obesity, insulin resistance, and type 2 diabetes [75,93,94,95,96,97]. Our findings indicate similar metabolic alterations in females who experienced high maternal objective hardship exhibiting elevated urinary glycine paired with decreased levels of serine, a constituent of glycine synthesis. Furthermore, these alterations in glycine and serine are also associated with the development of cardiovascular diseases, such as atherosclerosis, and Alzheimer’s disease, as these disorders share a similar etiology with metabolic syndromes [76,98,99]. These findings may provide a link between PNMS and a risk of developing these disorders later in life. In contrast, maternal subjective distress decreased serine paired with an increase in cystathionine in females. This suggests a potential vitamin B6 deficiency, that when severe, presents with seizures in younger individuals, and is implicated in depression, heart disease, cancer, and cognitive decline [100,101,102,103,104].

Females whose mothers experienced high objective hardship presented high potential impacts on the D-glutamate and D-glutamine metabolism. Glutamate is a vital excitatory neurotransmitter that plays a significant role in cognition, memory, learning, and synaptic plasticity. Excess extracellular glutamate can lead to neuronal damage and cell death via excitotoxicity and has been associated with diseases such as Alzheimer’s disease, chronic kidney disease, and schizophrenia [105,106,107]. Furthermore, increased intracellular glutamate leads to increased concentration of ROS [108]. Therefore, the upregulation of glutamate may play a role in the antioxidant pathways identified in females whose mothers experienced high objective hardship. An upregulation in urinary glutamine may implicate an impaired liver capacity to detoxify ammonia, thereby relying on glutamine synthesis. An oversaturation of the urea cycle may be caused by muscle protein breakdown due to impaired amino acid metabolism and increased proinflammatory cytokines [109,110,111]. Indeed, increased proinflammatory cytokines were reported previously in PNMS in the offspring of mothers who experienced the 1998 Quebec ice storm [112]. This is also supported by the possible dysregulation of amino acid metabolisms in females whose mothers experienced high objective hardship seen here through the glycine, serine, and threonine metabolism, arginine biosynthesis, and arginine and proline metabolism. Thus, females whose mothers experienced high objective hardship may present with impaired ammonia detoxification, thereby increasing their risk of kidney disorders, such as chronic kidney disease [113].

In contrast to females, males whose mothers experienced high objective hardship saw possible alterations in taurine and hypotaurine metabolism, lysine degradation, and pentose phosphate pathway; however, the potential impact on the pentose phosphate pathway was minimal. We observed no significant alterations in metabolic pathways in males whose mothers experienced high subjective distress, which is not consistent with previous findings in a similar disaster-related PNMS study [29]. This may be due to the use of only VIAVC significant bins, as only 14 bins, relating to 11 distinct metabolites, were utilized for pathway analysis. However, with only 14 bins a predictive accuracy of 87.2% was achieved. Lysine degradation presented as significant in males with high levels of maternal objective hardship due to the increased concentrations of 5-hydroxylysine and 2-aminoadipate. Elevations in hydroxylysines are found in almost all chronic degenerative diseases and are involved in the synthesis of 2-aminoadipate [114]. An increase in the concentration of 2-aminoadipate has been shown to occur due to the breakdown of lysine by oxidative stress. Furthermore, elevated levels of 2-aminoadipate due to the degradation of lysine have been implicated as a risk factor for the development of type 2 diabetes, atherosclerosis, and organic aciduria, leading to general metabolic acidosis, potentially triggering kidney abnormalities and liver damage [115,116,117,118]. These results may provide an additional mechanism for the elevated risk of chronic diseases in males who experience PNMS.

The dysregulation of aminoacyl-tRNA biosynthesis, a key mechanism of protein biosynthesis, and the galactose metabolism, a central part of cellular energy production, was potentially impacted in females prenatally exposed to either high objective hardship or subjective distress. Potential disruptions in the various energy metabolisms seen here may alter mitochondrial energy pathways, leading to an overabundance of oxidative stress compounds. This induction of oxidative stress can impact aminoacyl-tRNA biosynthesis by promoting mitochondrial tRNA mutations, common in type 2 diabetes mellitus [75,99,119]. Indeed, the alterations in protein biosynthesis and energy metabolism seen here support previous findings that adolescents exposed to high levels of objective hardship during the 1998 Quebec Ice Storm were at a higher risk of developing obesity [11,29,120]. Altogether, the present results suggest that PNMS increases ROS, altering antioxidant, energy, protein, and carbohydrate metabolisms. The gender differences in resiliency to ROS may explain the results and the increased risk of cardiovascular diseases in males.

Lastly, stress exposure frequently leads to adverse mental health, outcomes, which is also observed in offspring exposed in-utero to PNMS [121,122,123]. Results from this study support the increased risk of future adverse mental health outcomes. Taurine, upregulated in males whose mothers experienced high objective hardship, has been implicated in mental illness and is upregulated in individuals with autism and depression [124,125,126,127]. Previous research has also shown that glycine, serine, and threonine metabolism is associated with schizophrenia, autism, and depression [124,126,128]. Furthermore, oxidative stress provides another potential causal link between stress and mental health disorders [129]. These alterations present a prospective mechanistic role for adverse fetal-environment interactions in the development of adverse mental health outcomes later in life.

Metabolic profiling by ^1^H NMR spectroscopy in this study was able to distinguish unique metabolic profiles in males and females that potentially reflect their mother’s levels of objective hardship and/or subjective distress levels resulting from the 2011 Queensland Flood. Furthermore, considerable differences in the effects of PNMS on males and females were observed. Females displayed different oxidative stress and antioxidant responses as well as greater impacts on protein synthesis, carbohydrate metabolism, and energy metabolism than males. Females whose mothers experienced high objective hardship displayed the most dysregulation in these pathways due to an increase in glucuronidation and oxidative stress-related breakdown. However, males whose mothers experienced high maternal objective hardship were also observed to have potential impacts on energy metabolism and antioxidant pathways, suggesting a greater antioxidant response via the taurine and hypotaurine metabolism. Taken together, the present findings provide evidence that both maternal flood-related objective hardship and subjective distress increased oxidative stress in the ensuing offspring, altering key metabolic pathways. Our findings support the causal role of PNMS-related alterations in fetal development, thereby increasing the risk of offspring developing diseases related to oxidative stress. It is worth noting that the metabolic fingerprints identified in this study stem from a single timepoint, and therefore, have not yet been validated longitudinally. Furthermore, the risk of developing non-communicable diseases and mental illness due to PNMS is complex, and may be mitigated through social supports, coping strategies, and innate resiliency, among other factors [130,131,132]. Additionally, as this study only used 89 participants, the results from this study need to be verified in a larger population. Lastly, other factors not considered in this study may play a role in the present results, including environmental toxicant exposure due to the flood, lifestyle choices (diet, smoking, drinking, and exercise), mother’s health status, gestational age, and newborn health. These factors should also be accounted for when working with a larger population. Nonetheless, the identified metabolites may create new opportunities for personalized risk prediction and intervention for non-communicable chronic diseases in vulnerable populations.

## Figures and Tables

**Table 1:** Males: Statistically significant urinary metabolites identified by VIAVC or MW testing in the comparison of high and low maternal composite subjective distress and objective hardship in males. Significance was determined using a *p*-value equal to or less than 0.05. Metabolites are displayed in order of VIAVC *p*-value. Regulation relates to the high-strM Metabolites with more than one NMR resonance peak are represented in order of ppm as metabolite.1, metabolite.2, etc.

Group	Metabolite	NMR Chemical Shift Range of Bin (ppm)	VIAVC	Mann-Whitney U Test	Regulation
Male Subjective Distress (COSMOSS)	3-Hydroxyisovalerate	1.281–1.27	5.98 × 10^−122^	Not sig.	Up
	Indole-3-lactate.1	7.285–7.263	3.56 × 10^−82^	Not sig.	Up
	Creatine.1	3.947–3.929	1.32 × 10^−65^	1.04 × 10^−2^	Down
	Glycylproline	3.929–3.916	3.92 × 10^−53^	Not sig.	Down
	Methylguanidine	2.875–2.86	6.83 × 10^−48^	Not sig.	Down
	Citramalic acid	2.75–2.733	5.51 × 10^−37^	Not sig.	Down
	Creatine.2	3.045–3.03	2.19 × 10^−36^	2.57 × 10^−2^	Down
	Carnosine	2.671–2.662	3.09 × 10^−30^	Not sig.	Up
	Carnitine	3.227–3.215	1.20 × 10^−29^	Not sig.	Down
	3-Chlorotyrosine	7.129–7.116	3.25 × 10^−27^	Not sig.	Down
	3-Methylhistamine	3.723–3.712	1.40 × 10^−23^	2.17 × 10^−3^	Up
	Indole-3-lactate.2	4.383–4.33	2.10 × 10^−23^	2.04 × 10^−3^	Up
	(S)-3-Hydroxyisobutyric acid	1.116–1.105	2.98 × 10^−23^	Not sig.	Down
	O-Phosphocholine	3.241–3.227	4.17 × 10^−19^	4.45 × 10^−4^	Down
Male Objective Hardship (QFOSS)	Xanthurenate	7.105–7.095	1.41 × 10^−83^	Not sig.	Up
	Cysteine	3.011–2.996	2.01 × 10^−65^	Not sig.	Down
	3-Aminoisobutyrate.1	3.118–3.101	3.02 × 10^−48^	Not sig.	Down
	Creatine.1	3.947–3.929	5.98 × 10^−47^	Not sig.	Down
	Creatinine.1	3.066–3.045	1.06 × 10^−41^	Not sig.	Down
	Erythritol, Glycylproline	3.643–3.604	1.78 × 10^−35^	Not sig.	Up
	Gluconate.1	4.053–4.03	5.96 × 10^−34^	8.03 × 10^−3^	Up
	Dimethyl sulfone	3.164–3.156	2.37 × 10^−33^	Not sig.	Up
	Acetate	1.932–1.919	3.58 × 10^−30^	Not sig.	Up
	Creatine.2	3.045–3.03	5.73 × 10^−28^	Not sig.	Down
	5-Hydroxylysine.1	1.963–1.953	1.11 × 10^−24^	Not sig.	Up
	Creatinine.2	4.081–4.053	9.63 × 10^−24^	Not sig.	Down
	2-Methylbutyroylcarnitine.1	0.8867–0.8768	5.24 × 10^−23^	Not sig.	Down
	5-Hydroxylysine.2, Glutathione	2.963–2.942	3.40 × 10^−22^	Not sig.	Up
	Carnosine.1	2.702–2.684	2.02 × 10^−19^	Not sig.	Up
	5-Hydroxylysine.3	3.172–3.164	7.56 × 10^−19^	Not sig.	Up
	3-Aminoisobutyrate.2	1.196–1.185	1.49 × 10^−18^	Not sig.	Up
	4-Pyridoxic acid	2.358–2.339	7.62 × 10^−17^	Not sig.	Up
	Vanylglycol	3.375–3.366	1.19 × 10^−15^	Not sig.	Up
	Gluconate.2	4.157–4.134	1.66 × 10^−15^	4.05 × 10^−2^	Up
	Carnosine.2	2.671–2.662	2.03 × 10^−15^	Not sig.	Up
	2-Aminoadipate	2.26–2.247	2.10 × 10^−15^	Not sig.	Up
	Ribose	4.134–4.106	3.55 × 10^−14^	Not sig.	Up
	Allantoin	5.401–5.381	3.92 × 10^−14^	Not sig.	Down
	Taurine.1	3.448–3.437	5.86 × 10^−14^	Not sig.	Up
	Taurine.2	3.426–3.418	6.32 × 10^−14^	Not sig.	Up
	Citramalic acid	2.75–2.733	1.32 × 10^−13^	Not sig.	Up
	2-Methylbutyroylcarnitine.2	0.8768–0.866	1.82 × 10^−12^	Not Sig.	Down
	3-Phenyllactate	7.333–7.316	3.02 × 10^−12^	4.45 × 10^−2^	Down
	3-indoxylsulfate	7.513–7.495	2.10 × 10^−10^	Not sig.	Up
	Cysteine-S-sulfate	3.524–3.511	1.01 × 10^−09^	Not sig.	Down
	3,4,5-Trimethoxycinnamic acid, Kynurenine	6.815–6.776	1.39 × 10^−09^	Not sig.	Down

**Table 2:** Females: Statistically significant urinary metabolites identified by VIAVC or MW testing in the comparison of high and low maternal composite subjective distress and objective hardship in females. Significance was determined using a *p*-value equal to or less than 0.05. Metabolites are displayed in order of VIAVC *p*-value. Regulation relates to the high-stress group. Metabolite with more than one NMR resonance peak are represented in order of ppm as metabolite.1, metabolite.2, etc.

Group	Metabolite	NMR Chemical Shift Range of Bin (ppm)	VIAVC	Mann-Whitney U Test	Regulation
Female Subjective Distress (COSMOSS)	Proline.1	3.409–3.4	1.56 × 10^−99^	Not sig.	Up
	Lactose.1	4.699–4.676	2.63 × 10^−80^	1.45 × 10^−3^	Down
	Epinephrine	2.797–2.779	2.41 × 10^−77^	Not sig.	Down
	5-Aminolevulinic acid	2.806–2.797	6.22 × 10^−77^	1.35 × 10^−2^	Down
	Cysteine, Serine	3.966–3.947	1.68 × 10^−69^	2.50 × 10^−2^	Down
	Galactose	5.29–5.276	2.05 × 10^−50^	1.77 × 10^−3^	Down
	4-Hydroxyproline	3.498–3.489	1.74 × 10^−49^	2.89 × 10^−2^	Down
	Lactose.2	5.261–5.239	1.94 × 10^−24^	1.85 × 10^−2^	Down
	Tyramine, Carnosine	3.252–3.241	2.47 × 10^−23^	3.11 × 10^−2^	Up
	Sucrose	3.489–3.482	1.24 × 10^−22^	Not sig.	Down
	Cystathionine	2.208–2.198	8.34 × 10^−22^	Not sig.	Up
	2-Aminoadipate.1	2.26–2.247	5.47 × 10^−21^	Not sig.	Up
	Chlorogenate	5.357–5.352	1.11 × 10^−16^	2.15 × 10^−2^	Down
	5-Methoxytryptamine	6.972–6.964	4.92 × 10^−16^	4.12 × 10^−2^	Up
	Homovanillic acid	6.776–6.739	2.43 × 10^−15^	Not sig.	Up
	3-Chlorotyrosine	6.984–6.972	4.27 × 10^−14^	Not sig.	Up
	3-Nitrotyrosine	7.028–7.02	1.27 × 10^−13^	4.41 × 10^−2^	Up
	Indole-3-lactate	7.775–7.762	2.25 × 10^−13^	Not sig.	Up
	Proline.2	3.4–3.393	2.44 × 10^−11^	Not sig.	Up
	Pantothenate	0.9397–0.9273	8.19 × 10^−11^	Not sig.	Down
	Isobutyrate	1.085–1.075	1.50 × 10^−8^	Not sig.	Down
	2-Hydroxy-3-methylvalerate	0.8528–0.8431	3.12 × 10^−8^	Not sig.	Down
	Isoleucine	1.012–1.003	3.29 × 10^−8^	Not sig.	Up
	2-Aminoadipate.2	1.695–1.653	1.08 × 10^−6^	Not sig.	Up
	Pyroglutamate	2.391–2.385	1.14 × 10^−6^	Not sig.	Up
Female Objective Hardship (QFOSS)	Epinephrine	2.797–2.779	2.33 × 10^−61^	Not sig.	Down
	Cysteine, Serine.1	3.966–3.947	8.49 × 10^−53^	7.29 × 10^−3^	Down
	Glycine	3.586–3.564	3.61 × 10^−52^	Not sig.	Up
	Serine.2	3.869–3.863	4.24 × 10^−52^	Not sig.	Down
	(S)-3-Hydroxybutyric acid	1.254–1.245	1.55 × 10^−49^	Not sig.	Up
	Glutamine.1	2.163–2.152	1.11 × 10^−41^	3.89 × 10^−2^	Up
	Serine.3	4.002–3.994	8.30 × 10^−39^	Not sig.	Down
	Cysteine-S-sulfate	3.536–3.524	9.29 × 10^−37^	Not sig.	Down
	3-Chlorotyrosine	7.129–7.116	8.67 × 10^−32^	Not sig.	Up
	Isobutyrate.1	1.085–1.075	2.59 × 10^−30^	Not sig.	Down
	Erythritol.1	3.801–3.784	1.06 × 10^−29^	4.45 × 10^−2^	Down
	Theophylline	7.949–7.937	1.65 × 10^−29^	Not sig.	Up
	4-Hydroxyproline	3.498–3.489	3.55 × 10^−29^	Not sig.	Down
	Glucuronate	4.66–4.649	6.10 × 10^−27^	Not sig.	Up
	Erythritol.2, Lactose.1	3.643–3.604	5.15 × 10^−23^	Not sig.	Down
	3-Phenyllactate	2.887–2.875	8.49 × 10^−22^	Not sig.	Up
	Isobutyrate.2	1.075–1.065	7.82 × 10^−19^	Not sig.	Down
	Glutamine.2	2.474–2.465	4.10 × 10^−18^	Not sig.	Up
	Lactose.2	5.261–5.239	1.81 × 10^−17^	Not sig.	Down
	Isoleucine	1.036–1.012	1.02 × 10^−16^	Not sig.	Up
	Fucose	1.265–1.254	3.11 × 10^−16^	Not sig.	Down
	Phenylalanine	4.03–4.021	5.43 × 10^−13^	3.32 × 10^−3^	Down
	Symmetric dimethylarginine	2.85–2.817	2.32 × 10^−12^	Not sig.	Down
	Saccharopine.1	2.425–2.417	1.14 × 10^−11^	Not sig.	Down
	Sucrose	3.489–3.482	1.32 × 10^−10^	Not sig.	Down
	Maltose	5.407–5.401	4.81 × 10^−10^	Not sig.	Down
	Saccharopine.2	2.417–2.404	2.44 × 10^−9^	Not sig.	Down
	Proline	3.418–3.409	6.91 × 10^−9^	Not sig.	Up
	Ethanolamine	3.146–3.14	9.59 × 10^−9^	Not sig.	Up
	Glutamate	2.329–2.319	1.12 × 10^−8^	Not sig.	Up
	UDP-glucose	4.397–4.383	4.25 × 10^−8^	3.39 × 10^−2^	Down
	5-Aminolevulinic acid	2.806–2.797	7.56 × 10^−8^	Not sig.	Down
	Sarcosine	2.768–2.75	8.93 × 10^−8^	Not sig.	Down
	3,4-Dihydroxybenzeneacetic acid	6.739–6.715	2.07 × 10^−7^	Not sig.	Up
	Unidentified Metabolite @ 8.65 ppm	8.663–8.646	2.85 × 10^−6^	Not sig.	Down
	Malonate	3.133–3.118	3.07 × 10^−6^	Not sig.	Down
	Unidentified Metabolite @ 0.55 ppm	0.5574–0.535	3.29 × 10^−6^	Not sig.	Up
	N-Acetylglutamine	2.036–2.03	1.32 × 10^−5^	Not sig.	Up
	Arabinose	4.527–4.52	1.76 × 10^−5^	Not sig.	Up

## Data Availability

The data presented in this study are available on request from the corresponding authors. The data are not publicly available due to privacy reasons.

## Supplementary Materials

The following supporting information can be downloaded at: https://www.mdpi.com/article/10.3390/metabo13040579/s1, Table S1: Pathway topology analysis of statistically significant metabolic pathways generated utilizing metabolites identified as significant by VIAVC in the comparison of high and low maternal objective hardship for both males and females, and high and low maternal composite subjective distress for females. Analysis of maternal composite subjective distress for males is not reported, as there were no metabolic pathways identified as significant (*p*-value less than or equal to 0.05). Pathways are displayed by order of *p*-value. A −Log10(*p*) of 1.3 or greater is equivalent to a *p*-value of 0.05 or less.
