# Supplementary material for: Urinary 1H NMR Metabolomic Analysis of Prenatal Maternal Stress Due to a Natural Disaster Reveals Metabolic Risk Factors for Non-Communicable Diseases: The QF2011 Queensland Flood Study
Source: Metabolites. 2023 Apr 21;13(4):579. doi: 10.3390/metabo13040579 (PMC10145263; doi:10.3390/metabo13040579)
Supplement: Supplementary file 1 [file metabolites-13-00579-s001.zip › metabolites-2332041-supplementary.pdf]

Supplemental Table 1. Pathway topology analysis of statistically significant metabolic pathways generated utilizing metabolites identified as significant by VIAVC in the comparison of high and low maternal objective hardship for both males and females, and high and low maternal composite subjective distress for females. Pathway topology analysis of maternal composite subjective distress for males is not reported as there were no metabolic pathways identified as significant (p-value less than or equal to 0.05). Pathways are displayed by order of p-value. A -Log10(p) of 1.3 or greater is equivalent to a p-value of 0.05 or less.

Groups	Pathway Results	Raw p	-LOG10(p)	Impact	ID Metabolites
Male Objective Hardship (QFOSS)	Taurine and hypotaurine metabolism	0.0051158	2.2911	0.42857	L-Cysteine; Taurine;
	Pentose phosphate pathway	0.037427	1.4268	0.04712	D-Ribose; D-Gluconic acid;
	Lysine degradation	0.047378	1.3244	0.14085	L-2-Aminoadipate; L-Hydroxylysine;
Female Subjective Distress (COSMOSS)	Glycine, serine and threonine metabolism	0.00079075	3.102	0.21707	L-Serine; L-Cystathionine; L-Cysteine; 5-Aminolevulinate;
	Aminoacyl-tRNA biosynthesis	0.003291	2.4827	0.16667	L-Cysteine; L-Serine; L-Isoleucine; L-Proline;
	Galactose metabolism	0.0050899	2.2933	0.20208	Sucrose; Lactose; alpha-D-Galactose;
	Cysteine and methionine metabolism	0.009009	2.0453	0.2963	L-Cystathionine; L-Serine; L-Cysteine;
	Tyrosine metabolism	0.017523	1.7564	0.03714	L-Adrenaline; Tyramine; Homovanillate;
	Pantothenate and CoA biosynthesis	0.02603	1.5845	0.00714	Pantothenate; L-Cysteine;
Female Objective Hardship (QFOSS)	Aminoacyl-tRNA biosynthesis	1.22E-06	5.9132	0.16667	L-Phenylalanine; L-Glutamine; L-Cysteine; Glycine; L-Serine; L-Isoleucine; L-Proline; L-Glutamate;
	Glycine, serine and threonine metabolism	0.00026396	3.5785	0.55577	L-Serine; Glycine; Sarcosine; L-Cysteine; 5-Aminolevulinate;
	Glyoxylate and dicarboxylate metabolism	0.0024763	2.6062	0.14815	L-Serine; Glycine; L-Glutamate; L-Glutamine;
	Starch and sucrose metabolism	0.0039813	2.4	0.13303	Sucrose; UDP-glucose; Maltose;
	Nitrogen metabolism	0.0048415	2.315	0	L-Glutamate; L-Glutamine
	D-Glutamine and D-glutamate metabolism	0.0048415	2.315	0.5	L-Glutamate; L-Glutamine
	Ascorbate and aldarate metabolism	0.0088294	2.0541	0.5	UDP-glucose; D-Glucuronate;
	Galactose metabolism	0.012742	1.8948	0.15148	Sucrose; Lactose; UDP-glucose;
	Glutathione metabolism	0.014093	1.851	0.11182	Glycine; L-Glutamate; L-Cysteine;
	Porphyrin and chlorophyll metabolism	0.017032	1.7687	0.02799	Glycine; 5-Aminolevulinate; L-Glutamate;
	Arginine biosynthesis	0.026767	1.5724	0.11675	L-Glutamate; L-Glutamine;
	Arginine and proline metabolism	0.032014	1.4947	0.22479	Hydroxyproline; L-Proline; L-Glutamate;
	Pentose and glucuronate interconversions	0.042975	1.3668	0.20312	UDP-glucose; D-Glucuronate;
